# Enhanced heart failure, mortality and renin activation in female mice with experimental dilated cardiomyopathy

**DOI:** 10.1371/journal.pone.0189315

**Published:** 2017-12-14

**Authors:** Ranjana Tripathi, Ryan Sullivan, Tai-Hwang M. Fan, Dong Wang, Yao Sun, Guy L. Reed, Inna P. Gladysheva

**Affiliations:** 1 Departments of Medicine, University of Tennessee Health Science Center, Memphis, Tennessee, United States of America; 2 Department of Comparative Medicine, University of Tennessee Health Science Center, Memphis, Tennessee, United States of America; University Medical Center Utrecht, NETHERLANDS

## Abstract

Dilated cardiomyopathy (DCM) is the major cause of heart failure affecting both women and men. Limited clinical studies show conflicting data in sex-related differences in the progression of dilated cardiomyopathy and heart failure (HF) outcomes. We examined the comparative sex-related progression of cardiomyopathy and the development of HF (at 4, 7, 13 weeks of age) in a well-established, transgenic mouse model of DCM that recapitulates the progressive stages of human HF. By 13 weeks of age, female mice with DCM had more severe left ventricular systolic dysfunction, left ventricular dilation and wall thinning (P<0.001 for all) than age-matched male mice with DCM. Female mice also had greater lung edema (P<0.001), cardiac fibrosis (P<0.01) and pleural effusions, which were not rescued by ovariectomy. By comparison to DCM male mice at 13 weeks, these pathological changes in female mice with DCM, were associated with significant increases in plasma active renin (P<0.01), angiotensin II (P<0.01) and aldosterone levels (P<0.001). In comparison to DCM male mice, DCM female mice also showed differential expression of the natriuretic peptide system with lower corin and higher ANP, BNP and cGMP levels at 13 weeks of age. We conclude, that female mice with experimental DCM have an accelerated progression of cardiomyopathy and HF, which was not corrected by early ovariectomy. These alterations are associated with early renin activation with increased angiotensin II and aldosterone levels, and altered expression of the natriuretic peptide system.

## Introduction

Dilated cardiomyopathy (DCM) is the major cause of heart failure (HF) [1,2] that affects more than 60% of HF patients [1,3]. DCM affects both men and women [2,4] and sex-related differences in survival have been reported previously [2,4–8], but the data on disease incidence, outcomes and survival are inconsistent [9–14]. Whether sex-related variations in specific pathways alter the progression of cardiomyopathy or the development of HF remains poorly understood.

In humans, DCM is characterized by progressive enlargement of ventricles, fluid retention and loss of heart contractile function [1,2,4]. The heart muscle weakens and the main chamber (left ventricle) is unable to pump sufficient blood and oxygen to the organs [3]. Experimental data on diseases incidence, progression and HF outcomes in animal models are scarce. This experimental DCM mouse model appears to be the highly translationally relevant since the DCM mice are not only recapitulate pathological features of HF [15–18], but also pass through all stages of HF [19], similar to humans [20]. DCM mice develop progressive ventricular dilation with loss of systolic function, lung edema and death [15,17–19,21]. Female mice with DCM have shorter survival than males, which has been attributed to more severe impairments in mitochondrial function in females evident at 12 weeks of age [16].

The natriuretic peptide (NP) system and renin angiotensin aldosterone system (RAAS) play important roles in the pathophysiology of HF [22–26]. However, whether sex-related modulation of NP and RAAS affects the progression of DCM and HF is not clear. Activation of the RAAS leads to extracellular fluid and sodium retention, ventricular remodeling, left ventricular dysfunction and dilation; pharmacologic blockade of RAAS activity is widely used for HF treatment [27–30]. Under normal physiological conditions, RAAS action is counter-balanced by the NP system [31]. Natriuretic peptides oppose the effects of RAAS by increasing glomerular filtration rate, inhibiting sodium reabsorption and inhibiting renin and aldosterone release from the kidney [22,32].

Previous experimental studies have concluded that the net effect of estrogen is to suppress the renin-angiotensin system [33,34]; this suggests the hypothesis that female mice with DCM may be protected from RAAS activation and HF development in an experimental model of DCM. In this study we examined the progression of cardiomyopathy and HF over time, in female mice (with and without ovariectomy) by comparison to male mice with DCM. We monitored for the pathologic development of cardiomyopathy and HF (lung edema, cardiac dysfunction, cardiac remodeling) in relation to the activation of RAAS system and the expression of NP system components.

## Material and methods

### Mice

Animal studies were approved by the Animal Care and Use Committees of University of Tennessee Health Science Center and were performed in accordance with National Institute of Health (NIH) Guide for the Care and Use of Laboratory Animals. The progression of HF and cardiomyopathy were examined in female and male mice with or without DCM on a C57BL/6 background at 4, 7 and 13 weeks of age. DCM mice express a dominant-negative CREB transcription factor in cardiomyocytes and develop progressive DCM and HF [15,17–19,21]. The expression of dominant negative CREB transgene product was not different between sexes and the CREB transgene expression does not significantly alter the content of endogenous CREB protein in the heart between sexes [16].

Mice were housed in the same cage racks in AAALAC-accredited facilities and fed a normal chow diet with 0.3% sodium (EnvigoTeklad #7912) and their health and behavior were monitored daily by the animal facility staff and laboratory members. Mice with DCM of both sexes develop HF over time by experimental design. To prevent animal’s suffering, the following endpoint criteria were used in veterinary evaluation: weight loss (>20 percent), increased respiratory rate, and hunched posture. Mouse survival was determined by the reports of the animal facility technician who was blinded to genotype. Animals were reported deceased at daily health check prior to any endpoint criteria being identified; All 109 mice used in survival studies were found dead without using humane intervention (euthanasia). Total 216 mice were euthanized with isoflurane at 4, 7 and 13 weeks of age for blood and tissue samples collection; these mice were never demonstrated the endpoint criteria. The blood samples were collected by heart puncture into EDTA-aprotinin tubes to block proteolysis of targeted proteins. The blood samples were centrifuged at 3000 rpm for 20 min at 4°C, aliquoted, and stored at -80°C until analysis.

Systolic blood pressure (SBP) and diastolic blood pressure (DBP) were measured by a noninvasive tail-cuff method at 85–90 days old male and female mice with or without DCM (n = 26) (Kent Scientific, Torrington, CT; model XBP 1000).

### Echocardiography

Transthoracic echocardiograms were performed by an echocardiographer blinded to mouse genotype using a Vevo 2100 Imaging System (Visual Sonic Inc., Toronto, Canada) as we have described previously [17–19]. Hair from the ventral thorax was removed by chemical depilatory cream (Nair) one day before the echocardiographic studies. Briefly, mice were sedated with 1.5% inhaled isoflurane. Two-dimensional and M-mode images of the LV were obtained from the parasternal long-axis and short axis acoustic windows. The 2D-guided M-mode recordings were analyzed using Vevo LAB^®^ (version 1.7.1) software; left ventricular internal dimension at end systole (LVIDs), interventricular septal wall thickness in diastole (IVSd) and left ventricular posterior wall thickness in diastole (LVPWd) were measured on at least three cardiac cycles and averaged for each mouse. All measurements were performed using the leading-edge-to-leading-edge convention. The fractional shortening (FS, %) and ejection fraction (EF, %) were calculated according to standard equations.

### Lung edema analysis

Pleural effusions were assessed by necropsy analysis of the mouse thoracic cavity. Lung edema was assessed by the lung weight-to-body weight ratio (LW/BW%). The LW/BW% was calculated as right plus left lung wet weight divided by body weight as described previously [19]. The heart weight-to-body weight ratio (HW/BW%) was also calculated as an indicator of cardiac mass [17,18].

### Enzyme immunoassay for plasma proteins detection

Plasma corin, ANP (as N terminus-ANP), BNP (as C terminus-BNP), cGMP, angiotensin II and aldosterone levels were measured by enzyme immunoassays according to the manufacturer’s protocols (USCN Life Science Inc., China; Phoenix Pharmaceuticals, Inc.,Burlingame, CA; Enzo Life Science Inc.,Farmingdale, NY, Abcam Inc.,Cambridge, MA). Renin activities in plasma samples were measured by cleavage of a fluorescence resonance energy transfer substrate using a SensoLyte 520 mouse renin assay kit (AnaSpec, Fremont,CA) that allows the direct assessment of renin activity [34].

### Histological staining and analysis

Formalin-fixed (10% buffered formalin), paraffin-embedded heart sections (5 μm) were cut on slides and used for picrosirius red staining to analyze fibrosis. Slides were deparaffinized (Safeclear II, Fisher Diagnostics, Kalamazoo, MI) in a series of xylene solutions, hydrated in graded ethanol, and then stained with picrosirius stain (Abcam,Cambridge, MA). Slides were scanned by Aperio CS2 scanner (Aperio Technologies, Vista, CA) and images were captured using ImageScope (Leica Biosystems) software. Interstitial fibrosis was examined by collagen (red) positive area in 7–12 fields (scale bars, 200 μm) of left ventricular free wall and septum per heart by a blinded observer. Total interstitial fibrosis was quantitated as the percentage of the collagen (red) positive area/total myocardial area using Image Pro Plus 6.2 (Media Cybernetics, Bethesda, MD).

### Ovariectomy

Ovariectomy was performed in DCM female mice at 4 weeks of age prior to sexual maturation [35]. Mice were kept under 1–2%isoflurane anesthesia during surgical procedure. A single 1 cm ventral incision was made through the skin, muscle layer and body wall. Once exposed, the ovaries were clamped with a hemostat briefly to achieve hemostasis and removed. The body wall and muscle layers were sutured with 4−0 Vicryl and the skin was closed with surgical wound clips. Buprenorphine (0.1 mg/kg SC) was given at the time of recovery and every 12 h as needed up to 72 h post-surgery. Body temperature was maintained throughout the surgery using a heated surgical platform and a circulating hot water pad during recovery. Mice were returned to housing room after reaching full consciousness. Skin staples were removed 10–14 days post-surgery.

### Statistical analysis

Statistical analysis was performed with Graph Pad Prism 5.0 software (San Diego, CA). Survival was analyzed by the Kaplan-Meier method and the comparison of two groups was assessed by the log-rank test. Time-dependent differences between sexes were analyzed by a two-way ANOVA using the Bonferroni post-test correction. Differences among more than two groups were analyzed by one-way ANOVA at single time point (13 weeks) using a Neumann-Keuls correction. Differences were considered to be significant if the two-tailed P≤0.05. The number of animals (N) is indicated in the figures or legends. Data were expressed as mean ± SEM.

## Results

### Female mice have an accelerated decline in cardiac function

**Fig 1:**
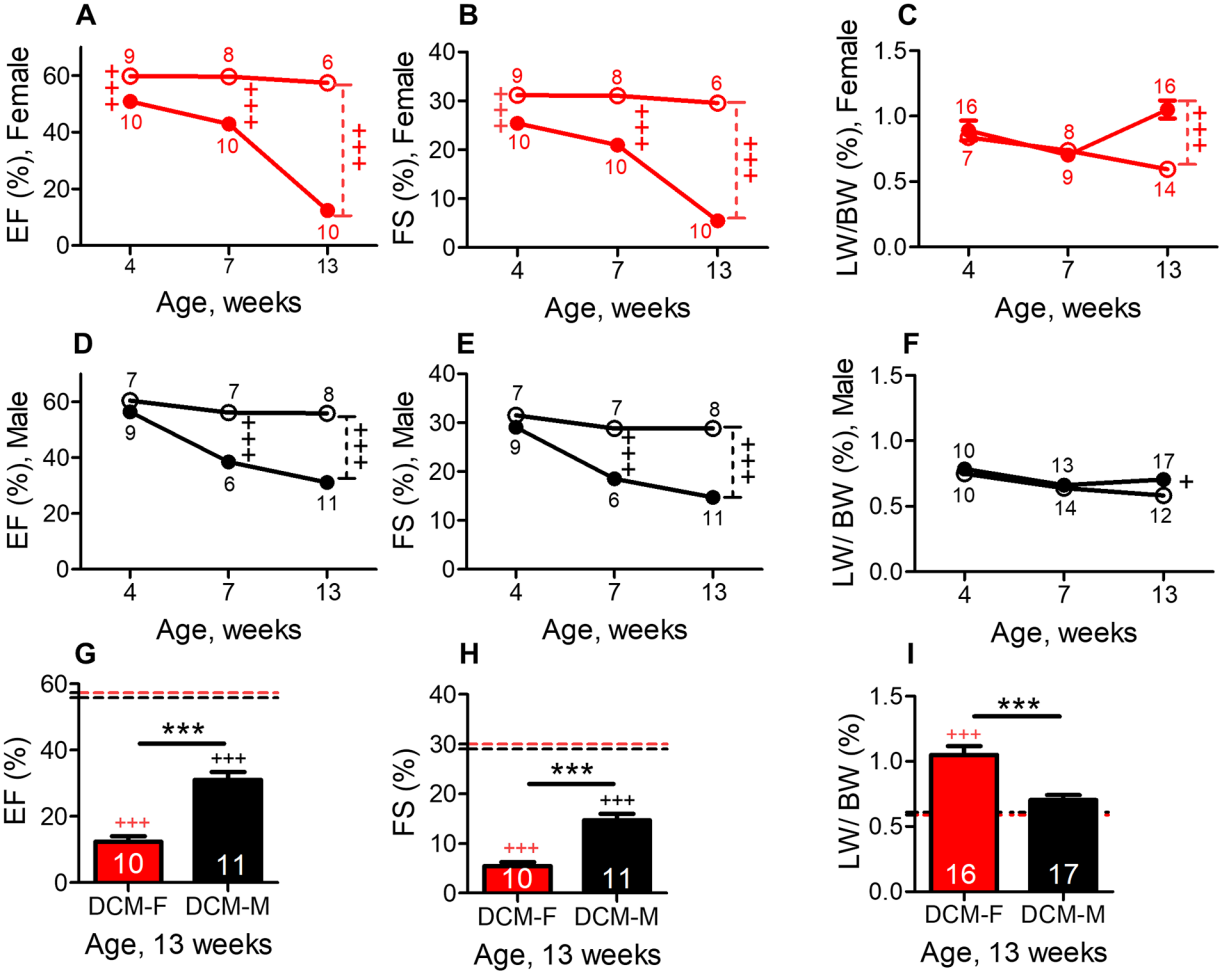
Female mice with DCM show accelerated declines in systolic function and increased lung water retention when compared with male mice with DCM. Age- related changes in ejection fraction (EF%) in female (A) and in male mice(D) fractional shortening (FS%) in female (B) and in male mice (E) LW/BW% in female (C) and in male (F) mice with and without DCM. Figs 1D, 1E and 1F were reproduced from our previously published data [19]. Female or male with DCM are indicated by closed symbols, female or male without DCM by open symbols. The number of animals per group at each time point is shown. Age dependent changes in both sexes were analyzed by two-way ANOVA. At 13 weeks of age, changes in EF% (G) FS% (H) and LW/BW% (I) in female and male mice with or without DCM. DCM-F (DCM female, red), DCM-M (DCM male, black), dotted red (female) and black (male) line represent mean baseline levels in mice without DCM. The number of DCM mice is indicated. Differences among groups at 13 weeks were analyzed by one-way ANOVA. ***P<0.001 (DCM female vs DCM male). ^+++^P<0.001, and ^+^P<0.05 (DCM vs. WT mice).

We compared cardiac systolic function in DCM female mice to WT female controls. In DCM female mice, EF% (Fig 1A) or FS% (Fig 1B) were mildly decreased at 4 weeks of age and progressively declined by more than 75% at 13 weeks (EF = 12±2% vs. 57±1%, Fig 1A, P<0.001 and FS = 5.5±.7.0% vs 30±0.7%, P<0.001, Fig 1B). In DCM male mice, a less marked, time-dependent decline in systolic function was evident in comparison with wild-type controls (Fig 1D and 1E), as we have previously described [19].

The sex-based differences in cardiac systolic function in female vs. male mice with DCM were examined. The EF% was similar in female vs. male mice with DCM at 4 weeks (51±2% vs 56±1%) and 7 weeks (43±2% vs 38±2%), but it decreased nearly 2.5-fold in female vs male at 13 weeks of age (EF%; 12±2% vs 31±3%, Fig 1G). The FS was also markedly reduced in female vs male mice at 13 weeks (5.5±0.8% vs 15±1%, Fig 1H, P<0.001). Age (P<0.001) and sex (P<0.001) both are crucial determinants of systolic function (EF% and FS%) in DCM mice, as analyzed by two-way ANOVA.

At 13 weeks of age, female mice with DCM had significantly greater left ventricular dimensions than male with DCM (LVIDs, 4.3 vs 3.9 mm, S1C Fig, P<0.05). At this age, the interventricular septum (IVSd = 0.5 vs0.7mm, P<0.001) and posterior wall (LVPWd = 0.5vs 0.7mm, P<0.001) were significantly thinner in female vs male mice with DCM (S1F and S1I Fig). In comparison to their sex matched WT controls, the thickness of IVS and LVPW were significantly decreased (13 weeks) in DCM female mice (P<0.001; S1D and S1G Fig) but only slightly reduced in DCM male mice (IVSd = NS;S1E Fig; LVPWd = P<0.01;S1H Fig). These echocardiographic measures demonstrate that female have severely reduced left ventricular function with greater ventricular dilation and more pronounced wall thinning than male mice with DCM.

### Female mice develop greater lung edema

The development of HF is associated with pleural effusions and increased lung water retention or lung edema in patients [22] and in mice with DCM as previously reported [17–19]. In DCM female, at 4 and 7 weeks of age, there was no sign of lung edema and pleural effusions, but 13 weeks DCM female showed a significant increase by 77% (P<0.001) in LW/BW% (lung edema) and pleural effusions in comparison with WT female controls (Fig 1C). A less significant change (4–13 weeks) in LW/BW% was evident in DCM male mice in comparison to WT male controls (Fig 1F) as described [19].

Importantly, DCM female mice also showed a significant increase (by 50%) in LW/BW% (Fig 1I, P<0.001) and pleural effusions (female = 3 out of 17 animals vs male = 0 out of 12 animals) in comparison with DCM male mice at 13 weeks. LW/BW% were analyzed by two-way ANOVA in DCM mice of both sexes; age (P<0.01) and sex (P<0.01) both were significant modulators of lung weight.

### Female mice show enhanced pathological cardiac remodeling

**Fig 2:**
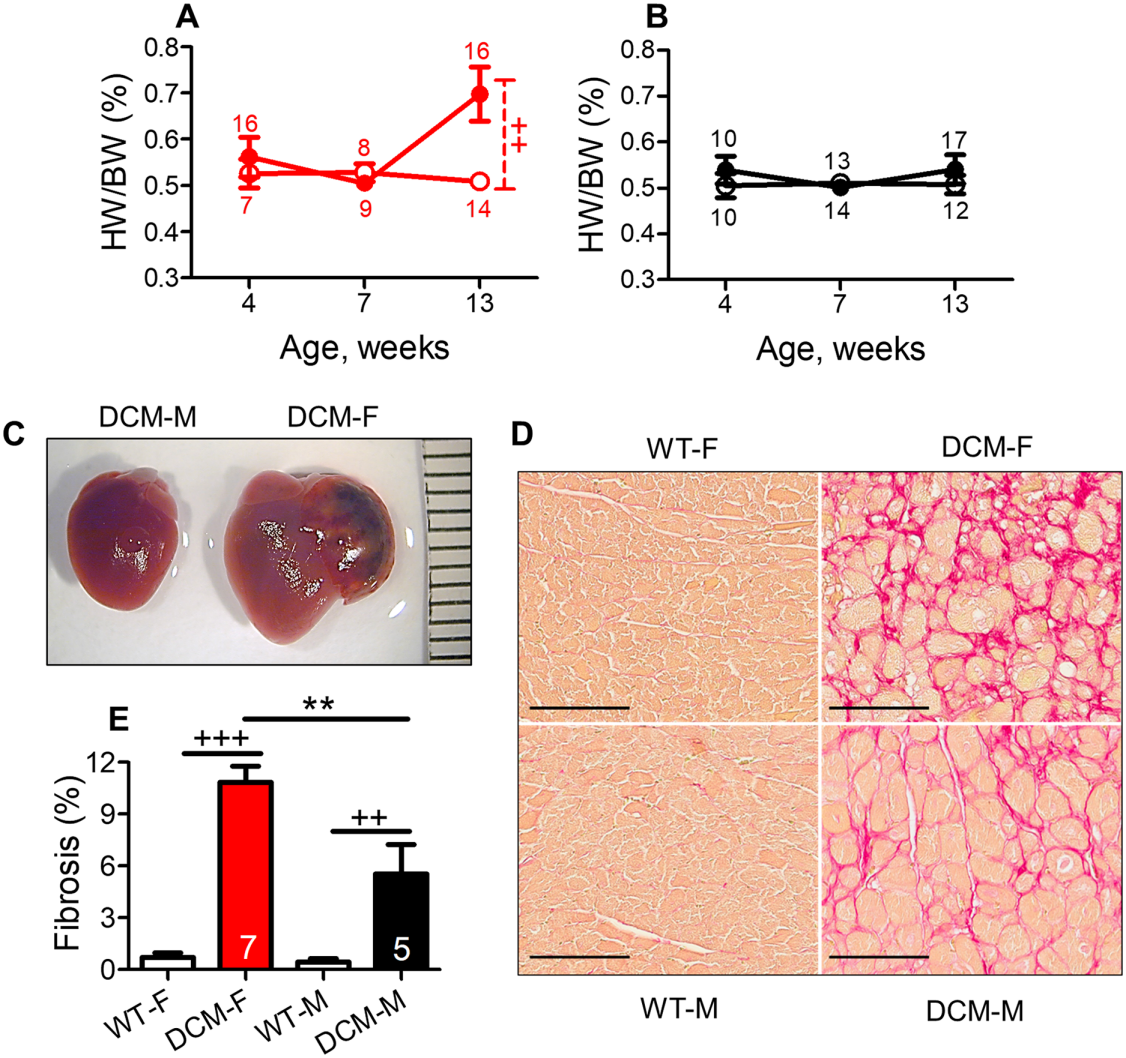
Sex differences in cardiac fibrosis and hypertrophy in DCM mice. (A) Heart weight to body weight ratio (HW/BW%) in female and (B) in male DCM and WT mice. Female or male mice with DCM are indicated by closed symbols, female or male WT controls mice by open symbols. Number of animals per group at each time point is shown. (C) Representative (scale bars, mm) image shows gross heart morphology of female and male mice with DCM at the age of 13 weeks. (D) Representative picrosirius red-stained images (scale bars, 100 μm) show collagen from heart tissue of DCM and WT, female and male mice of 13 weeks of age. Red color indicates collagen deposition. (E) Interstitial percent fibrosis was calculated in measurements from 6–12 images (scale bars, 200 μm) of the left ventricle and septum, n = 4–8 per group. WT female and WT male (open bar), DCM female (red bar) DCM male (black bar). **P<0.01 (DCM male vs. DCM female), ^+++^P<0.001, ^++^P<0.01 (DCM vs. WT) mice.

We determined cardiac remodeling by measuring HW/BW% and cardiac fibrosis. By comparison to their sex matched WT controls, both female (P<0.001) and male (P<0.01) mice with DCM showed increased cardiac fibrosis (Fig 2D and 2E) at 13 weeks of age. Although male DCM mice did not show any difference in HW/BW% as compared to their WT control males (Fig 2B), DCM female mice showed significantly increased HW/BW% in comparison to WT female control (Fig 2A, P<0.01) at 13 weeks.

We compared sex-based differences in HW/BW% and fibrosis in DCM female vs DCM male mice at 13 weeks of age. HW/BW% was increased in DCM female vs. DCM male (Fig 2A and 2B) and that is also evident by morphologically enlarged heart seen in DCM females (Fig 2C). DCM mice had pathologically enlarged and dilated heart chambers as shown in Fig 2C. DCM females had four chamber dilation with significant left ventricular enlargement when compared to DCM males at 13 weeks of age (Fig 2C). Left atrial enlargement with thrombosis was often seen in females with DCM, but not in males (Fig 2C).

We determined age-dependent changes in HW/BW% by a two-way ANOVA in female and male mice with DCM; this analysis showed that the age was a significant (P<0.05) modulator of cardiac mass in DCM mice. Cardiac fibrosis was significantly enhanced in female vs. male mice with DCM as assessed by picrosirius red staining for collagen (P<0.01, Fig 2D and 2E) at 13 weeks.

### Female mice show enhanced early activation of the RAAS

**Fig 3:**
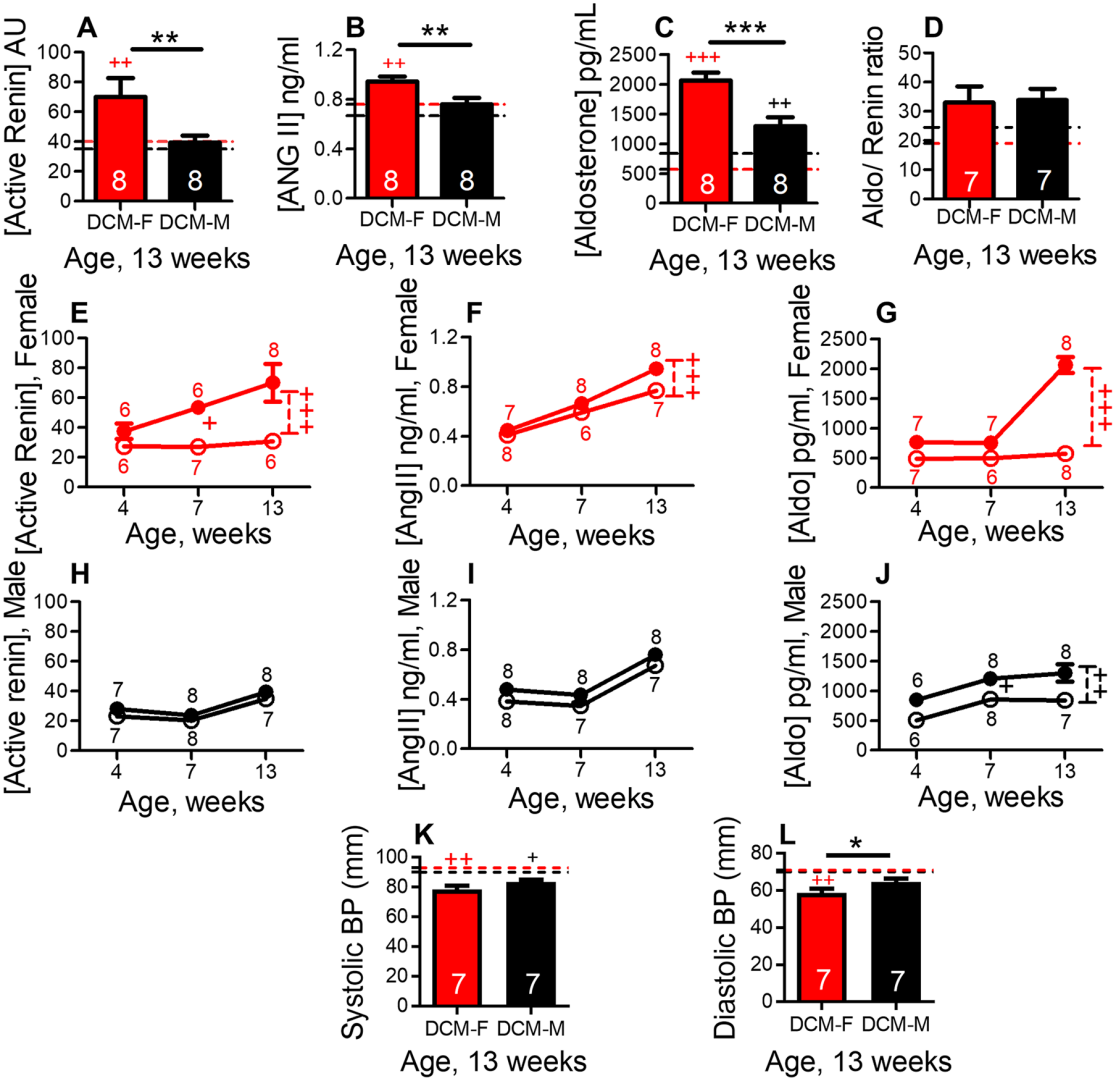
Sex differences in the activation of RAAS axis in mice with DCM. Plasma levels of (A) active renin (B) angiotensin II and (C) aldosterone in female and male DCM and WT control mice, at 13 weeks, determined by ELISA. (D) Aldosterone to active plasma renin ratio in DCM and WT mice. The number of DCM mice is indicated. Female and male base line levels were indicated by red and black dotted line respectively (WT mice, n = 6–8 per group). Plasma active renin levels (arbitrary units) in female (E) and in male (H), angiotensin II plasma levels in female (F) and in male (I), Aldosterone plasma levels in female (G) and in male, DCM and WT control mice (J). The number of animals per group at each time point is shown. Female and male mice with DCM (closed symbol) female and male WT mice (open symbol). Systolic blood pressure (K) and diastolic blood pressure (L) in female and male, DCM and WT mice. The number of DCM mice is indicated, WT mice, n = 4–8 per group. ***P<0.001, **P<0.01, *P<0.05 (DCM female vs. DCM male). ^+++^P<0.001, ^++^P<0.01, ^+^P<0.05 (DCM vs WT).

Activation of RAAS,with elevated plasma angiotensin II and aldosterone, promotes HF, lung edema and myocardial fibrosis [22,28,36]. The RAAS is activated by renin followed by angiotensin II production and aldosterone release [27]. By comparison to their WT controls, plasma active renin levels (P<0.01), angiotensin II (P<0.01) and aldosterone levels (P<0.001) were increased in DCM female (Fig 3A–3C). However, DCM males showed only a significant elevation of aldosterone (Fig 3C, P<0.01) levels as compared to their WT controls.

We compared sex-based differences in plasma RAAS components in DCM female vs DCM male mice at 13 weeks of age. Plasma active renin levels were significantly increased in female vs male mice with DCM (Fig 3A, P<0.01). Angiotensin II (P<0.01) and aldosterone (P<0.001) plasma levels were also increased in female vs male with DCM (Fig 3B and 3C). There was no significant difference in the aldosterone to renin ratio in female and male mice with or without DCM (Fig 3D). At 13 weeks of age, systolic blood pressure (SBP) was unchanged in DCM female vs DCM male mice (Fig 3K) and diastolic blood pressure (DBP) was reduced in DCM female vs DCM male mice (Fig 3L, P<0.05).

We determined the longitudinal changes in RAAS activation at 4, 7 and 13 weeks of age and its relationship with the development of HF. In comparison to their WT controls, female DCM mice showed an early increase in active renin plasma levels at 7 weeks (Fig 3E, P<0.05) that further increased at 13 weeks (Fig 3E, P<0.001). Angiotensin II (P<0.001) and aldosterone (P<0.001) plasma levels were significantly increased only at late stage (13 weeks), as significant HF developed in DCM females (Fig 3F and 3G). However, DCM male mice showed a significant increase (P<0.001) in aldosterone only as compared to their WT controls but not in renin or angiotensin plasma levels (Fig 3H–3J). We found that in DCM mice, age (P<0.0001) and sex (P<0.001) both are significant modulators of renin plasma activity and angiotensin II plasma levels while aldosterone levels are modulated only by age (P<0.0001) when analyzed by two-way ANOVA.

### Female mice show increased activation of the NP system

**Fig 4:**
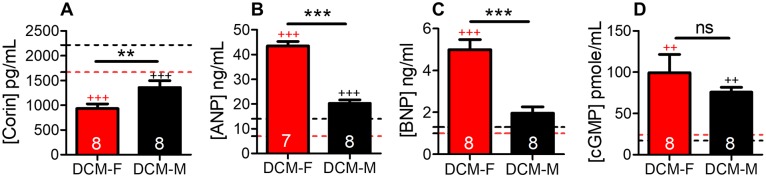
Natriuretic peptide activation in female mice. Plasma levels of (A) corin (B) ANP (C) BNP and (D) cGMP in DCM and WT, female and male mice, at 13 weeks, determined by ELISA, data represent mean± SE. DCM female (red bar), DCM male (black bar). Data for male mice have been reported [19]. The number of DCM mice per group is indicated. Female and male base line levels were indicated by red and black dotted line respectively, n = 6–8 per group. ***P<0.001, **P<0.01 (DCM male vs. DCM female). ^+++^P<0.001, ^++^P<0.01 (DCM vs. WT), ns = non significant.

Low corin levels are found in HF patients [37–39]and experimental DCM with reduced systolic function [17,19]. Corin plasma levels were significantly reduced (P<0.001, Fig 4A) and, ANP and BNP plasma levels were increased in DCM female (P<0.001, Fig 4B and 4C) by comparison to WT female controls (Fig 4A). In comparison to WT male controls, DCM male showed reduced corin plasma levels (P<0.001, Fig 4A), and increased ANP plasma levels (P<0.001, Fig 4B) as we described [19]. cGMP plasma levels were significantly increased in both female and male mice with DCM vs their sex-matched WT controls (Fig 4D; P<0.01).

We determined sex related differences in plasma levels of corin, ANP and BNP. There was a significant decrease in corin (P<0.01) and an increase in ANP, BNP (P<0.001) plasma levels in DCM female mice in comparison to DCM male mice (Fig 4A–4C). cGMP plasma levels were non-significantly increased in DCM female vs DCM male mice at 13 weeks of age (Fig 4D).

### Ovariectomy does not rescue female mice from declining systolic function, HF or mortality

**Fig 5:**
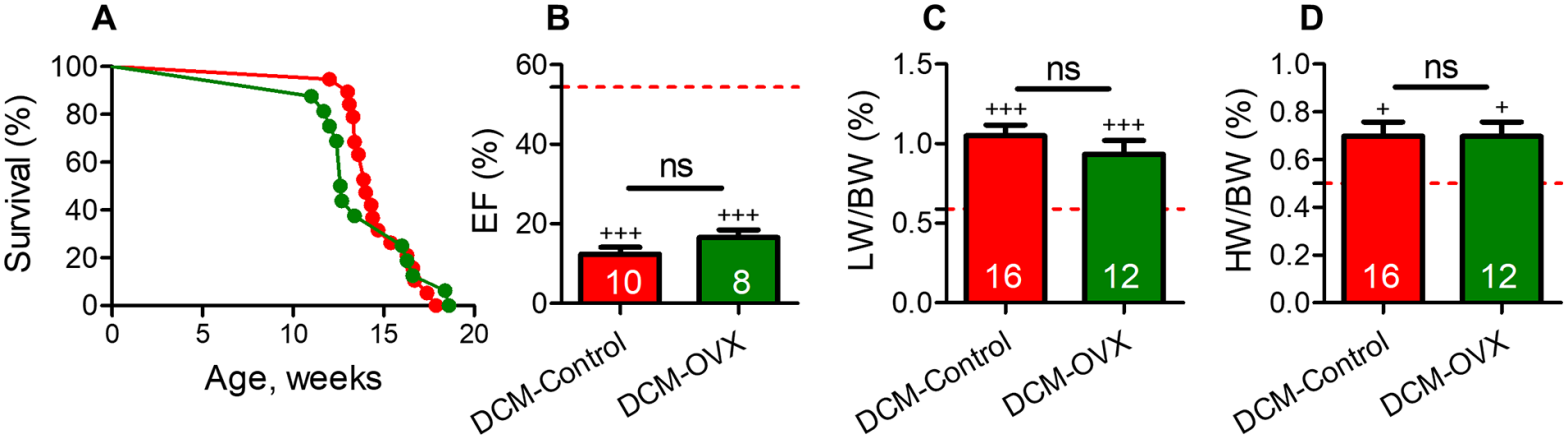
Ovariectomy does not significantly alter survival, systolic dysfunction or and HF development in female mice with DCM. (A) Kaplan–Meier survival curves of DCM- OVX vs. DCM non-OVX control female. DCM non-OVX control female (red, n = 27), DCM-OVX female (green, n = 22), WT female (red dotted line) (B) Systolic function (EF%) (C) LW/BW% and (D) HW/BW% were indifferent in DCM-OVX vs DCM non-OVX female. The number of DCM mice is indicated. Data represent mean± SE, ^+++^P<0.001, ^+^P<0.05 (DCM vs. WT mice).

Consistent with previous reports in another strain of mice [16], we confirmed that females had a markedly diminished survival vs. male mice with DCM (median survival—female, 13.7 vs male, 20.1 weeks, P<0.001). The promoter for angiotensinogen (renin substrate and precursor of angiotensin II) is directly controlled by estrogen through estrogen response elements [33,34] which suggests that estrogen may modulate systolic dysfunction, HF and mortality in female mice with DCM. Although ovariectomy (OVX) reduces estrogen levels [40] it did not significantly prolong survival in DCM-OVX females in comparison to DCM-control (non-OVX) females (median survival, 13.8 vs. 13.9 weeks, Fig 5A). Heart systolic function (EF%) was similarly reduced in DCM-OVX vs DCM control females when compared at 13 weeks of age (Fig 5B). In addition, in mice with vs. without ovariectomy, there were no significant differences in pleural effusions (3 out of 17 vs 4 out of 10 mice), LW/BW% (Fig 5C), HW/BW% (Fig 5D), active renin plasma levels (P>0.05, n = 8 per group) and cGMP plasma levels (P>0.05, n = 10 per group).

## Discussion

These data show that females have an accelerated progression of DCM and increased mortality in a translationally relevant, experimental mouse model of DCM. Female mice with DCM have accelerated systolic dysfunction, ventricular dilation, increased lung edema and cardiac fibrosis, and reduced survival as compared to male. These differences are associated with marked early activation of the RAAS, depressed corin levels with profound elevations of ANP, BNP and cGMP in DCM females.

Female mice with DCM progressed through the ACCF-AHA Stages of HF [20] faster than male mice with DCM [19]. At 4 weeks of age, males had Stage A HF (risk of DCM without decline in systolic function), but females at this age already showed a significant decline in EF%, corresponding to Stage B of HF. At 13 weeks, males had Stage C HF, whereas females showed Stage C-D (terminal HF and death). At 13 weeks of age, female mice had physiological signs of HF such as lung edema, pleural effusions, heart dilation and increased myocardial fibrosis. Plasma HF biomarkers such as ANP, BNP and cGMP were also elevated. There were also notable sex-based differences in EF%, FS%, LVIDs, IVSd, LVPWd, LW/BW% and plasma HF markers consistent with the accelerated mortality in female vs male mice with DCM.

Enhanced RAAS activity is associated with clinical signs of HF in humans including fluid retention, pulmonary venous congestion, pleural effusions, heart dilation and myocardial fibrosis [22–27,41]. Active renin exclusively controls the first rate limiting step in RAAS cascade i.e. conversion of angiotensinogen to angiotensin I. The early (at 7 weeks) and sustained elevation of active renin (13 weeks) observed in female mice with DCM may have contributed to the more severe interstitial fibrosis, ventricular dilation, HF and greater mortality observed in these mice [24,42]. At 13 weeks, female with DCM showed increased active renin, angiotensin II and aldosterone levels vs male with DCM. These data are consistent with clinical studies showing that women have higher aldosterone levels and are more sensitive to aldosterone-induced ventricular remodeling as compared to men [41,43,44]. The degree of increase in plasma renin activity and plasma aldosterone level correlates with prognosis in patients with left ventricular HF [22,45–47]. The finding that aldosterone/active renin ratios were similar in both sexes of mice with or without DCM, suggests that aldosterone production remained under the control of the active renin cascade (vs. autonomous production). Measures of kidney function (plasma BUN and creatinine) were within normal range in DCM vs WT mice of both sexes, excluding abnormal renal function as a cause of RAAS activation [17,18]. RAAS components are regulated by sex hormones and modulated in a sex-dependent manner under pathological conditions [33,34]. However, the effect of female sex on RAAS modulation may be complex, as ovariectomy did not influence plasma active renin, survival, systolic function, lung water retention, and cGMP levels in ovariectomized DCM vs. non-overiectomized DCM female mice.

Overexpression of cardiac corin improves contractile function and prolongs survival in DCM mice in part through reduction of myocardial fibrosis [17]. We found that female mice with DCM had lower corin levels and increased cardiac interstitial fibrosis vs. male mice with DCM. Further studies are required to determine whether there are sex-related differences in the molecular composition of this fibrosis with regards to specific collagens and other extracellular matrix proteins. However, by comparison to sex-matched control mice, ANP and BNP peptides are much higher in female than in male mice with DCM. These data are in agreement with lower plasma corin levels in women than men in HF patients [38]. Impaired pro-ANP processing has been implicated in humans with decompensated HF with low corin levels and, it may contribute to HF in DCM mice as well [37,48]. The NP system is protective in the development of HF, it suppresses the RAAS components and thus mitigate fluids retention and cardiac fibrosis. In DCM mice, females showed enhanced RAAS activity, which likely contributes to their early demise. Although RAAS is elevated it did not increase systolic or diastolic blood pressure in female mice with DCM. NP system components such as ANP and BNP levels were increased in these females, which might be a compensatory response to attenuate the deleterious effects of RAAS activation, but insufficient to halt the development of HF in females.

Sex-related differences have also been noted in mice with cardiac overexpression of PDGF-C and the cardiac alpha1B adrenergic receptor [49,50]. PDGF-C female mice develop severe DCM and die of HF, while DCM male mice show progressive hypertrophy without HF symptoms [49]. Cardiac alpha1B adrenergic receptor overexpression leads to early death in female vs male mice, but no differences in other HF parameters were noted [50]. In contrast, with the DCM model used in the present study, mice from both sexes shown a similar progression of DCM-HF, but at different rates, which uniquely recapitulates all of the stages of human HF (Stage A-D) [19].

It has been reported that females are more likely to develop HF in the setting of hypertension, diabetes or after the acute phase of myocardial infarction [16]. While it is important to consider the translational limitations of murine studies, in this experimental model, female sex also altered the development of HF and the progression of cardiomyopathy. The pathogenesis appears complex, as ovariectomy did not rescue these female mice from HF nor did affect mice with another model of DCM [51]. This suggests that the sex-related difference in DCM mice may not be simply attributable to hormonal status, but rather by the effects of sex chromosomes on differential expression of myocardial and non-myocardial genes [52–55].

The increased renin levels in DCM females may be driven by sex-related differences in upstream pathways such as the kinin-kallikrein system or factor XII activity [56,57]. In addition, sex-related changes in local cardiac RAAS or natriuretic peptide activity may also modulate cardiac remodeling [25,30,58–61]. An alternative axis of the RAAS, which comprises ACE2, neprilysin, Ang-(1–7) might also play a role. DCM mice also show early evidence of mitochondrial dysfunction (4 weeks), though sex-related differences in mitochondrial function were not found until 12 weeks in females, which is after they develop significant systolic dysfunction and HF [16]. Further studies are required to assess the extent to which these sex-related differences in the progression of systolic dysfunction and HF are generalizable to other types of cardiomyopathies (e.g., ischemic cardiomyopathy) and, whether they can be modulated by agents that alter renin, estrogen, mitochondrial function or other components of the RAAS or natriuretic peptide pathways to reduce HF and mortality.

## Conclusions

Our study clearly demonstrates that, in an experimental model of DCM, female sex is associated with accelerated decline in systolic function and, the development of HF, which is accompanied by early and sustained activation of systemic RAAS as well as increased plasma natriuretic peptide levels. Further clinical studies may be warranted to gain insight into how sex affects the progression of HF and treatment response in patients with DCM, with special reference to the RAAS and natriuretic peptide pathways.

## Supporting information

S1 Fig Increased heart dilation in female mice with DCM. (DOCX)
