# Supplementary material for: Enhanced heart failure, mortality and renin activation in female mice with experimental dilated cardiomyopathy
Source: PLoS One. 2017 Dec 14;12(12):e0189315. doi: 10.1371/journal.pone.0189315 (PMC5730114; doi:10.1371/journal.pone.0189315)
Supplement: S1 Fig — (DOCX) [file pone.0189315.s001.docx]

**Supporting information**

**S1 Fig: Increased heart dilation in female mice with DCM.**

**
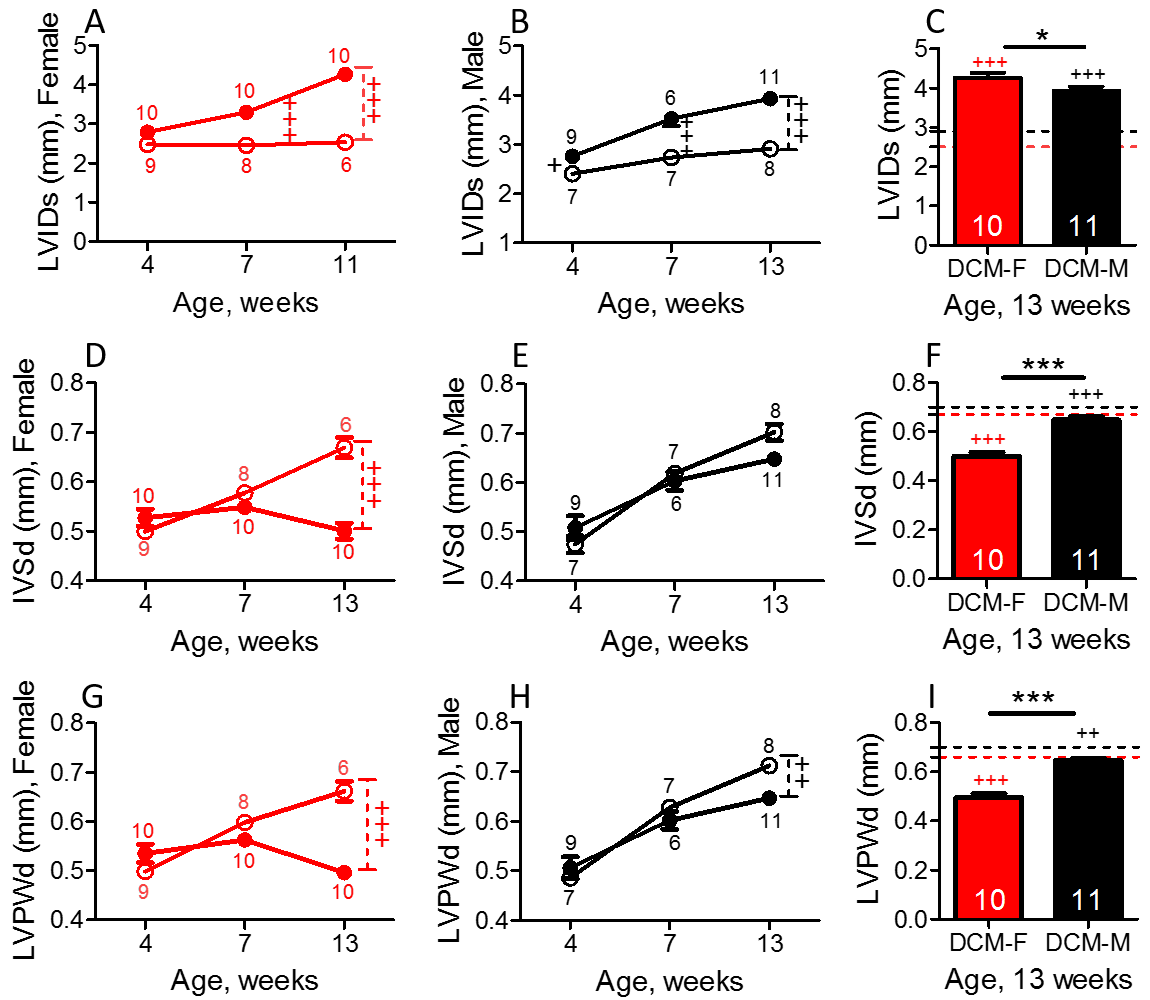
**

**S1 Fig:** Age related changes in left ventricular internal dimension at end systole (LVIDs) in female (A) and in male (B), Interventricular septal thickness at end diastole (IVSd) in female (D) and in male (E), left ventricular posterior wall at end diastole (LVPWd) in female (G) and in male (H) mice with or without DCM. The number of animals per group at each time point is shown. Female or male with DCM (closed symbol), female or male WT mice (open symbol). Data represent mean± SE, ^+++^P<0.001, ^+^P<0.05 (DCM vs. WT) mice. (C-F-I) At 13 weeks of age, LVPWd (C), IVSd (F) and LVIDs (I) in female and male with or without DCM. The number of DCM mice is indicated. DCM-F (DCM female), DCM-M (DCM male). WT female (red) and WT male (black) dotted line, ***P<0.001, *P<0.01 (DCM female vs. DCM male).
